# The Relation of Medicine to the Natural Sciences

**Published:** 1905-09

**Authors:** J. Michell Clarke


The Bristol

Medico-Chirurgical Journal.

"*Scire est nescire, nisi id me
Scire alius sciret.*"

SEPTEMBER, 1905.

INDEX
THROUGH

b239

### THE RELATION OF MEDICINE TO THE NATURAL SCIENCES.

Presidential Address at the Annual Meeting of the Bath and Bristol Branch of the British Medical Association, June 28th, 1905.

BY

J. MICHELL CLARKE, M.A., M.D.Cantab., F.R.C.P.

IF there is one feature more than another which distinguishes the civilisation of to-day from that of former ages, it is the rapid rise out of comparatively insignificant beginnings of the vast and imposing structure of modern science. The latter part of the nineteenth century especially is characterised by the remarkable developments that have taken place in the natural sciences and in scientific methods of thought, and it is safe to assert that of the learned professions none has advanced more rapidly, or shown greater changes in both its theory and practice, than medicine.[1] To no other profession has so wide a vista of future extension been opened up.

[1] I use the term medicine in its wider and proper sense to comprise both medicine, surgery, and all their branches.

This fortunate condition we owe to the great advance of modern science, which has extended the scope of the healing art, widened its interests, and brought it into touch with almost every form of scientific activity. We have only to consider modern surgery in order to realise the great gulf, the absolute difference in kind, which divides the medicine of the present day from that of even so recent a time as one hundred years ago. Science has entered so fully into our lives, has become such an intimate part of them, and we have come to regard this so much as a matter of course, that we rarely stop to think over the great changes that it has wrought.

The practice of medicine and surgery now consists essentially of the application to our art of the discoveries made in physics, chemistry, electricity, and biology. We might define present-day medicine as science applied to the study and treatment of disease. Not only is this true of practical methods, but thought and opinion have also been modified to an extent that we hardly realise. It is difficult to put ourselves into the mental attitude of our predecessors towards the problems of life, but I shall probably be correct in saying that the theory of evolution has effected no less striking a change in philosophy, in our views of life, and of the nature of the universe, than applied science has in our daily practice. We may, as Dr. Payne pointed out in his Harveian Oration for 1896, regard this modern advance in scientific knowledge as the latest of the several revivals that have occurred in preceding ages of the Greek spirit of inquiry at first hand into the laws of nature. In our own profession this principle of direct observation and experiment was first acted upon by Hippocrates and the Hippocratic physicians, and is exemplified in the remark of Aristotle, that "of doctors those who practice their art in a scientific spirit begin the study of medicine by working at natural science." The history of medicine is one of periods of inactivity, of torpor and stagnation, alternated with periods of advance. These latter have always been marked by a recrudescence of the Greek method of experimental investigation into natural phenomena. The periods of stagnation have equally been remarkable for absence of direct observation, for wealth of theory, and of stereotyped systems of medical practice. More than once, as for instance in the days of Galen, then again in the time of the circulation of the Arabian treatises on medicine, imperfect embodiments of the Greek spirit as they were, and, later still, when at the opening of the Renaissance large numbers of the original Greek manuscripts were brought into Italy, we owe a period of progress to the rediscovery and reperusal of the Greek masters themselves, and are thus directly indebted to their writings for a fresh step forwards. Far more important, however, than the actual facts and observations contained in their works is the gift we owe to them for laying down, once for all, the true principles on which alone a sound advance could be made. The discoveries of modern medicine and the enormous strides that it has made are not chiefly to be attributed to the invention of any new principle on which work should be based, but rather to the application to present-day problems of principles already established from of old with the aid of greater wealth of appliances, due to the infinitely greater extension of the allied sciences. Perhaps the new principle which most concerns our art, and which, although dimly foreseen in some speculations of the ancient philosophers, was never realised by them as a working force in biology, is that which underlies the doctrines of evolution and advance by natural selection. It is somewhat strange that this, one of the greatest contributions of modern science on its speculative side, has so far borne little fruit in the field of medical research. We can hardly say that the Darwinian theories have been worked out in relation to disease. This is partly due to the complex nature of the problems involved, partly also, I think, to the insufficient biological training which the majority of doctors receive. Consequently the purely biological aspect of medicine receives little consideration, although there must be much to be learnt in this respect, which would greatly help to elucidate the problems of disease as it affects different races.

The story of the relation of medicine to science in former ages, down, we may say, to the modern era, is briefly a history of the gradual rise and growth of science out of medicine through the labours of the medical profession. One of the chief glories of the history of medicine lies in the many important discoveries that have been made in pure science by medical men. I do not here allude to those who were medical men by accident, as it were, who soon abandoned their profession for more congenial occupation in the natural sciences—on such grounds we might as well claim poetry as a product of medicine because Keats was a doctor—but to men who, though engaged during most or the whole of their lives in the arduous pursuit of medicine, yet kept alight the torch of science and passed it on to their successors, leaving us for emulation their great example.

This condition of things was partly a consequence of the fact that down to the modern era men mostly studied the natural sciences as an approach or introductory study to medicine, as is inferred in the sentence of Aristotle I have already quoted. In Greek times there was nothing exactly corresponding to modern science; the natural sciences comprised descriptive botany and biology, with a certain amount of anatomy founded on dissections of animals, and a still smaller knowledge of physiology derived from the apparent uses of organs as shown by anatomical observations. Chemistry was unknown, and physics also in the modern sense of the term. The Greek study of the laws of physics, although in some directions it carried them far—Democritus, for instance, went very near to anticipating the atomic theory—was chiefly concerned with transcendental or speculative inquiries as to the nature and origin of matter, and as to the structure of the universe. These systems of physics and correlated metaphysical speculations did not greatly influence medicine, so that it escaped in these times an overloading with theory. Hippocrates and his followers kept to the sound paths of anatomical, pathological, and clinical observation. Hence medicine in its early days avoided the fate which awaited it in the Middle Ages, when it was divorced from experiment and observation, and held fast in the bonds of tradition and the preconceived theories of the schools.

During long subsequent ages medicine preserved the same relation to such science as was known, and with few exceptions the workers at science were medical men. In the early Renaissance the renewed study of science was the outcome of the revival of Greek learning, which arose from the passage into Italy of teachers from Constantinople, carrying with them manuscripts of the Greek writers on natural science. The scientific revival at this time accordingly followed the lines of the ancient Greek authors, and was practically confined to biology and anatomy. However, the study of anatomy, and of a certain amount of physiology in correspondence with it and derived mainly from anatomical observations, was carried by the aid of fresh investigations to a higher level than had ever been previously attained. Pathological observations on the dead body began to be made again, and the relation of *post-mortem* changes to symptoms during life to be traced out. Soon afterwards came the great discoveries of Harvey, who started anatomical and physiological researches on the right path, which they have ever subsequently followed, by insisting upon the necessity and demonstrating the value of the experimental method of inquiry, and incidentally almost at once placed anatomy and physiology on a sounder basis.

Hitherto, as we have said, medical progress had been wholly on the ancient lines of biological study. Long before this, however, the beginnings of the mystical study of alchemy in Alexandria had foreshadowed the rise of a new science, that of chemistry; but it is not until Paracelsus (A.D. 1493) that medicine was at all influenced by the beginnings of this science. Fantastic charlatan as in some respects he was, Paracelsus was the first to attempt, however imperfectly, to apply the then scanty knowledge of chemistry to the practice of medicine, and was the precursor of that iatro-chemical school which subsequently attained to so great an influence. The views of Paracelsus regarded diseases entirely in their relation to drugs, and had thus nothing in common with the traditional Galenical teaching of medicine, or with that of the anatomical school which succeeded it.

Sir Michael Foster points out that alchemy did not affect medicine earlier, because the alchemists who sought for the philosopher's stone and the elixir of life were not, as a rule, doctors, and, moreover, often carried on their work in solitude far apart from medical schools and universities. Hence "a certain antagonism arose between this nascent science of chemistry and the older biological studies which formed the basis of medical education. The latter was the heritage of a a long-established, powerful profession; the former was the product of amateurs, of the efforts of scattered independent workers, and as such was despised by professional men."[1]

Through this long period, then, science is mostly in debt to medicine for any advance that was made, and for some time still this remains true, but to a progressively less degree, as we now come with the origin of the sciences of physics and chemistry in the first part of the seventeenth century to the beginning of the modern era, in which the bulk of scientific work ceases to be done by medical men.

During the whole of the seventeenth and eighteenth centuries, however, considerable contributions to science continue to be made by physicians labouring side by side with workers in pure science. Now science begins to repay her debt to medicine, and to repay it a thousandfold, so much so that we shall find that in future we have to look to science for the source of the chief advances in our art. In connection with the early history of physics and its application to physiology the illustrious names of Galileo, Descartes, and of Borelli, the friend of Malpighi, cannot be passed without mention, and in chemistry that of Van Helmont, especially in relation to his work on fermentation. The new discoveries in physics and chemistry enabled these sciences to be employed in the elucidation of physiological problems, as in the work done on respiration by the English school of the latter half of the seventeenth century—that school, of which we have reason to be proud, which includes Boyle, Hooke, Lower, and Mayow—and as in the researches of Stephen Hales on the physics of the circulation, and thence to be applied to some extent, though as yet to a very limited one, to medical practice. Passing over the intervening period of steady progress in physics and chemistry during the eighteenth century, we come at the end of this century to the celebrated discovery of Galvani (1791). Let us not forget that Galvani

[1] Michael Foster, *Lectures on the History of Physiology*, Camb. Univ. Press, 1901.

as a Professor of Anatomy and Physiology belonged to our profession, for it is hardly too much to say that the splendid achievements of modern electrical science come in no small degree from his first physiological experiments, partly indirectly, indeed, through his controversy with Volta, which led to the discovery by the latter of the voltaic pile and of the production of electric currents by the action of fluids upon metals.[1] From this time onwards the relation between medicine and science undergoes an entire change. The increasing complexity and range of scientific inquiry made it impossible for the worker in science to be at the same time engaged in the practice of physic, and moreover to an ever-increasing extent led him to specialise in one subject. To achieve distinction or to advance knowledge, he began to find that he must limit his work to one branch of science. Hence we no longer find science an avenue to medicine, but that it has become an end in itself, and the names illustrious in science cease, with rare exceptions, to be those of medical men. At the same time medicine itself is about to enter upon a career of rapid and unprecedented development, largely to be attributed to that great growth of scientific knowledge which placed so many new resources at its disposal.

In many branches of medicine science has given us exact knowledge, results which can be weighed and measured, and which can be calculated upon with certainty, as a substitute for empiricism. It has made it possible, although unfortunately to a still too limited extent, to predict that given certain conditions certain results follow. We have seen that as the domain of science extended greater efficiency necessarily entailed more strict specialisation on the part of the workers, and this same tendency has shown itself as much or more in our own art. The infinitely more extensive, and at the same time more accurate, knowledge in the strict fields of medicine and surgery implies that less time can be devoted by medical men to general scientific studies. In this respect I doubt whether medicine can retain in the future the position that it has held in the past. There can be no doubt that the average level in scientific knowledge of the medical man of the future will be higher than

[1] McKendrick, *Life in Motion*, 1902.

it has ever been before, because of the better training that is given in the sciences that bear directly upon medicine. That contributions to pure science of the same excellence and of the same extent and variety as those made in the past will ever again be produced by medical men is not probable. But, on the other hand, compensation may be found for this loss in the wide and engrossing nature of the problems opened up for investigation in the field of pure medicine, by the application to its theory and practice of the discoveries made in other branches of science. There is no longer need for the doctor to seek outside his own profession for subjects of scientific inquiry; he can find in his own proper work subjects in which he may confidently expect to advance knowledge, and these neither limited in scope nor tending to narrow his mind, but rather to bring him into close relation with the widest problems of biology. The pursuit of medicine, practised in the right spirit, is one of the few which enables its followers "to see life steadily, and see it whole."

It is the gradual emergence of medicine from the empirical to the scientific stage that has changed so greatly the position of the doctors of the twentieth century from those of former times, and it is from this point of view that we can estimate best the advantages which we possess as compared with our predecessors. I may be pardoned for quoting in this context a well-known passage of Bacon, because it brings out so clearly the change in our profession in these respects. After remarking on one drawback of our calling that "in the opinion of the multitude, witches and old women and impostors have had a competition with physicians," a statement which unfortunately for us, and still more unfortunately for the lay public, is almost as true to-day as in his time, he goes on to say, "Therefore I cannot blame much physicians that they use commonly to intend some other art or practice, which they fancy, more than their profession. For you shall have of them antiquaries, poets, humanists, statesmen, merchants, divines, and in every one of these better seen than in their profession." This passage well exemplifies the change which has come over medicine as a profession, and has raised it again into a calling of wide intellectual interest. It seems to me that it also shows the point of difference between the doctors of our own day and those before the dawn of the modern era, and that this difference lies in the fact that the advance of medicine as a science, by giving the doctor fresh powers, has also given him confidence in his ability to heal and to increase knowledge, and that it was the absence of this hope of advancing the science and art of his own profession that drove the physician of Bacon's age to seek relief in other pursuits.

Of late years we have heard it said with increasing frequency that the learned physician of cultivated tastes will shortly become extinct. If it is meant by this that physicians will no longer, in Bacon's phrase, be better antiquaries, poets, or humanists than doctors, it is hardly to be regretted. After all, a physician, however eminent he may be in other branches of knowledge, should be learned as a physician first, and erudite in other ways afterwards. But fortunately the very nature of the medical calling brings the doctor into such close touch with all sorts and conditions of men, and with so many different forms of human activity, as to ensure that it will always contain men of wide culture. Possibly the physician of the future will attain to culture by ways which diverge from the traditional paths, his erudition may be chiefly in the natural sciences, that is to say in the science and knowledge of his times.

Of the way in which modern science may open out in a totally unforeseen direction a new branch of learning, and with it a new insight into disease and a new hope of progress in the healing art, the study of bacteriology offers a notable example. Passing over the philosophic interest of the discovery of forms of life so infinitesimally small, of a world of minute organisms hitherto undreamt of, the history of bacteriology strikingly illustrates how far-reaching and momentous a scientific discovery, at first sight of limited scope, may be. Who could have predicted that the work of Pasteur on ferments would lead to the elucidation of the true nature of infective disease, and still more remarkable would be the foundation of a new surgery, so extraordinarily successful in its results that it may well claim to be one of the chief scientific triumphs of the end of the nineteenth century. Great as the achievements of modern surgery and the benefits that it has conferred on mankind have been, the advance in the knowledge of infective diseases and of the means by which immunity from them can be secured is no less striking and promises to be as fruitful in beneficial results. It is difficult to realise that more has been learnt of the nature of infective disease in the last thirty years than during the long ages through which they have been known to mankind; and not only so, but a line of investigation has been started which has by no means come to an end, for before this chapter in the history of medicine is closed we may confidently expect that the mode of exterminating these disorders will have been effectually learnt.

The reproach has been laid against medicine that during the long period between the Hippocratic School and the time of Sydenham it departed from the rule of the former of carefully observing and recording individual cases. Although this will always be a necessary part of medical investigation, and the more fruitful in proportion to the closeness with which clinical observation is kept in touch with the science of the day, yet the advance made in this way is necessarily slow, and the experience of modern times has shown that carefully devised scientific experiments are a necessary adjuvant to it, and lead through wider generalisation to more rapid progress. Further, it has been found that the exhaustive study of special branches of medicine has been rewarded not only by the increased light thrown upon some dark corner of our art, but by fresh additions to the whole realm of medical science.

We have thus briefly retraced the path which medicine has followed in its gradual evolution from empiricism towards the position of an exact science. Can we yet say that it has taken the last and greatest step and attained this position, and, if so, what is its place amongst the exact sciences? The answer to the latter part of this question is fairly obvious, it must be as a branch of biology that medicine takes its place as a science. But instead of the problems dealing with the conditions of survival, increase, and growth with which biology is mainly concerned, medicine is occupied with the departures from the normal physiological standard, with the phenomena of decay or threatened dissolution. Disease not being an entity in itself we can most usefully conceive of it as a retrogression from the current standard of a healthy living activity. It is as a branch of biology that medicine deals with the results of the constant struggle between man on the one hand and the organisms that war against his existence on the other. So that medicine has to solve and to meet the problems connected with the survival of the dominant race—man—in his struggle with the forces that make for his extinction. We often forget, but should be constantly reminded, that in this internecine struggle nature carefully provides for the survival and growth of the lowest as well as of the highest forms of life. Blind and undiscriminating, she gives an equal chance to all. Hence it has always seemed to me that the "vis medicatrix naturæ," if it is meant by that term, as is so often implied in the common use of it, that nature benevolently works to protect and guard the highest organism—man—is a misnomer. Hence again, though we should all deprecate an unnecessary interference with "natural" processes of recovery from disease, and extended pathological knowledge has both informed us of the course of such processes and warned us of the importance of "non nocere," the doctrine of the "vis medicatrix naturæ" interpreted in the vulgar way leads to a sort of nihilism in medicine, which paralyses further advance in the art of healing. At the most all that the "vis medicatrix" can mean with regard to man and his departures from health is that in the course of ages his organs and tissues have attained to a position of equilibrium in working which is consonant with his best activity, and that when this equilibrium is disturbed by the incidence of disease there is in the majority of cases a tendency for it to be re-established.

Returning now to inquire into the conditions which must be fulfilled to constitute medicine an exact science, we find that, roughly stated, they are the following:—(1) The causes of disease must be known; (2) the precise alterations produced in the anatomical and chemical constitution of the body, and the exact organs and tissues affected must be ascertained; (3) the course which a disease runs and the results, if it is one which causes as sequelæ, temporary or permanent changes in organs and tissues, and in their secretions, must be determined; and (4) we should be able, given the conditions, to reproduce the disease at will. It is enough to constitute medicine as an exact science if we can satisfy these conditions for some diseases only. If in other words there is a sufficient knowledge of the general laws which control, and of the phenomena which characterise, certain diseases, to enable us to lay down general principles on which those still imperfectly known can be worked out in a scientific manner.

The history of medicine in recent years gives many striking instances in which diseases imperfectly known or even new to us have been investigated with complete success by the application of methods founded on general principles established by observation and experiment in similar affections. What better examples could be taken than the recent discoveries in malaria and in the less known tropical diseases, such as the disorders caused by trypanosomes.

With regard to the exhaustive investigation of a disease in the ways indicated above, this has of late years been accomplished in many instances. Turning to the infective disorders, and taking for instance anthrax or tetanus, we know the cause, the nature of the poisons at work, the anatomico-pathological changes produced in the organs, the course of the disease, and, in the case of tetanus, the mode and exact seat of the peculiar combination of the toxin with the cytoplasm of certain highly-specialised cells. We can reproduce the disease, and not only produce it, but so regulate the dose of the poison as to give a mild or severe attack; and, lastly, we can confer immunity against an attack by methods the principles of whose action is understood. In these infective disorders then, which form so large and important a branch of medicine, the advances in knowledge that have been made are so great as to constitute alone a well-founded claim for medicine as an exact science.

Perhaps, in nervous diseases, our acquaintance with the seat and nature of the anatomico-pathological lesions and with the finer changes in the protoplasm of cells is more complete than in any others. This knowledge has been acquired by utilising the extreme degree to which differentiation of function has been carried in the gradual evolution of the nervous system. The progress that has been made in the knowledge of the structure and function of this part of the body during the last generation is remarkable. But though the mode of action of certain poisons and toxins has been ascertained and the selective affinity of some of them for definite tracts and centres has thrown much light on the action of poisons in general, we are very much behindhand in our knowledge of the exact causes of nervous diseases. If we could make one step forwards, and recent work has given us fresh hope of this, we should fill a most important gap in scientific medicine—I mean if the organism of syphilis were discovered and its life-history known. If in this one disease we could work out the life-history of the organism and ascertain its chemical products, we should, by combining these fresh acquisitions with the already acquired accurate knowledge of the anatomical lesions, not only obtain a complete picture of the course of a most important disease, but incidentally get fresh light on the conditions underlying other nervous affections. It seems to me that we are close to the time when our knowledge of nervous disease will give medicine another title to be an exact science.

To take another example, and this time from the chemical side of medical work. No better example of the replacing of empiricism by scientific experiment could be given than is afforded by modern pharmacology in its investigation of the relation between chemical constitution and physiological action, and of the physiological effects of introducing various chemical groups into complex organic compounds whose physiological action is known. Then the recent experiments on digestion have taught us that cells, differing little in morphological characters, may show an unexpected differentiation of function. Recognition of functional differentiation of this kind is of the very essence of exactness in clinical work, and these experiments afford good evidence that parts of organs hitherto regarded as practically identical, may in reality carry on distinct and highly specialised activities.

In spite of these many and great advances, we have, however, still to deal in too large a field of disease with the uncertainty of result, which from a scientific standpoint is the bane of clinical medicine. Hence, to quote again from the passage of Bacon which I cited above, it is still true of our position as medical men "that the physician . . . hath no particular acts demonstrative of his ability, but is judged most by the event; which is ever but as it is taken: for who can tell if a patient die or recover, . . . whether it be art or accident. And therefore many times the impostor is prized, and the man of virtue taxed."

Many factors contribute to this uncertainty of result in actual practice. One of them is to be assigned to what is commonly known as the "idiosyncrasy" of the patient, but may be better termed the variable reaction of the individual, an unknown quantity for which large allowance has to be made in practice, and which often comes to baffle the best directed efforts at treatment and the most carefully-considered prognostications. Possibly to some minds, with a natural bent for speculation and theoretic inquiry, the very uncertainty of the issue in the individual possesses a charm in itself, as a food for thought and philosophic questioning. But however fascinating in the theory of medicine the problem of individual variation may be, it is anything but pleasing in the daily practice of our profession. We must long for the time when we shall be able to do our clinical work with exactness, and to look with confidence for precision of result. Another and still more potent cause of failure of our best directed efforts lies in the extreme complexity of the problems often presented in clinical medicine. There are causes at work which we fail to recognise, or we are misled by certain symptoms to attach undue importance to some minor factor, and to overlook the true cause of disease, either because at the time of investigation it may not be giving rise to clear signs, or, although present, their significance is not appreciated. The variation of the pathological processes that take place during the course of a long and complicated disease is also difficult to estimate aright. It results that when the work has been done in the most accurate and careful way possible we are confronted with a mortifying failure because the value of a certain factor or factors has not been correctly calculated. Such are two of the most obvious conditions which have in all times, and do now, render the practice of clinical medicine unsatisfactory from the standpoint of the scientific worker and expose the practitioner to unmerited reproach.

The remedy is to be sought in the increasing application of scientific methods to medicine, and it is to modern science that we must look to remove such obstacles from the path of medical progress. After all, when we speak of idiosyncrasy as altering the ordinary course of a disease we are but exposing our own ignorance. Diseases, like all other natural phenomena, move by exact laws, and the individual variations which now baffle us would appear, if our views were wide enough and our knowledge deeper, but as expressions of the action of these general laws.

In closing this inadequate summary of the relation between medicine and science, I may allude to one danger which it seems to me may impede our future advance, and that arises from the increasing specialisation which appears to be the necessary correlative of latter-day progress in all branches of science. The day of the encyclopædist in science seems to have passed. It is impossible for even a Darwin or a Kelvin to be in the first rank in more than one or two branches of it. This development of specialisation may lead to such an increase of technicality in methods of work and in the terms in which they are described as to make it barely possible for the workers in one branch of science to thoroughly appreciate what is being done by those engaged in another, so as to be capable of turning recent discoveries to account in their own special province.

From this cause there is a danger that medicine should again become too stereotyped, and thereby lose that close connection and interdependence with other sciences which have been so laboriously built up during the past three hundred years, and from which it has derived such enormous advantage. Should this unfortunately occur, it seems to me that, in spite of all the great advances that have been made, there might again ensue one of those sterile periods in our art in which once more "systems," with their arid and barren theories, would take the place of that constant scientific observation which is essential to true progress. It may seem impossible now that we have gone so far that our practice should become retrocedent, but it must be remembered that in former times men have been equally well satisfied with the extent of their knowledge. For instance, in the Roman civilisation of the age of the Antonies, which offers so many striking resemblances to our own era, a very high degree of material prosperity was reached. Men talked just as readily as we do now of the resources of modern civilisation, and probably the troups of students who followed Galen from bedside to bedside of his patients would have scouted the idea that their knowledge of medicine left much to be desired.

The remedy lies in constant and strenuous exertion to keep the science and art of medicine in close touch—no easy task—with every advance in biology, chemistry, and physics, that as science continually extends her beneficent domain, and as "the old order changeth, yielding place to new," medicine may, by taking and holding fast what is of permanent good in both new and old, fight with ever-increasing success against disease and death.

